# Supplementary material for: Water pumping in mantle shear zones
Source: Nat Commun. 2017 Jun 8;8:15736. doi: 10.1038/ncomms15736 (PMC5472755; doi:10.1038/ncomms15736)
Supplement: Supplementary Information — Supplementary Figures, Supplementary Tables and Supplementary References [file ncomms15736-s1.pdf]

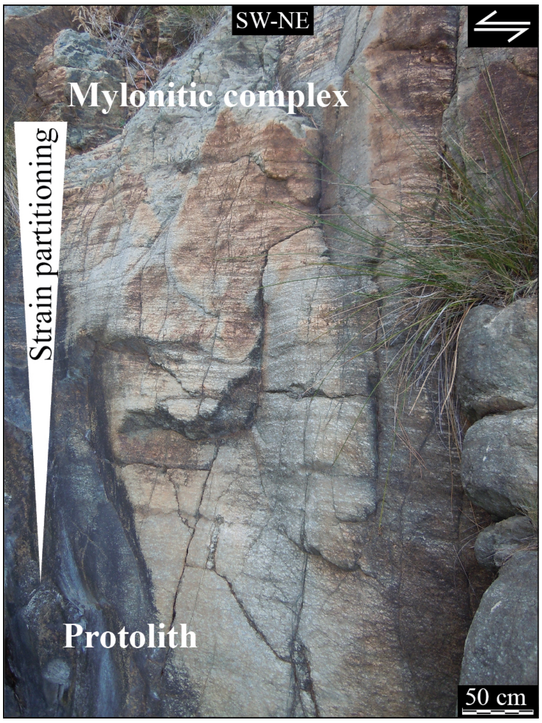

Supplementary Figure 1 | Strain partitioning at the edge of mylonitic complexes. From the protolith to the mylonitic complexes, the strain partitioning between the protomylonite layers (dark) and the mylonite layers (bright) progressively strengthens.

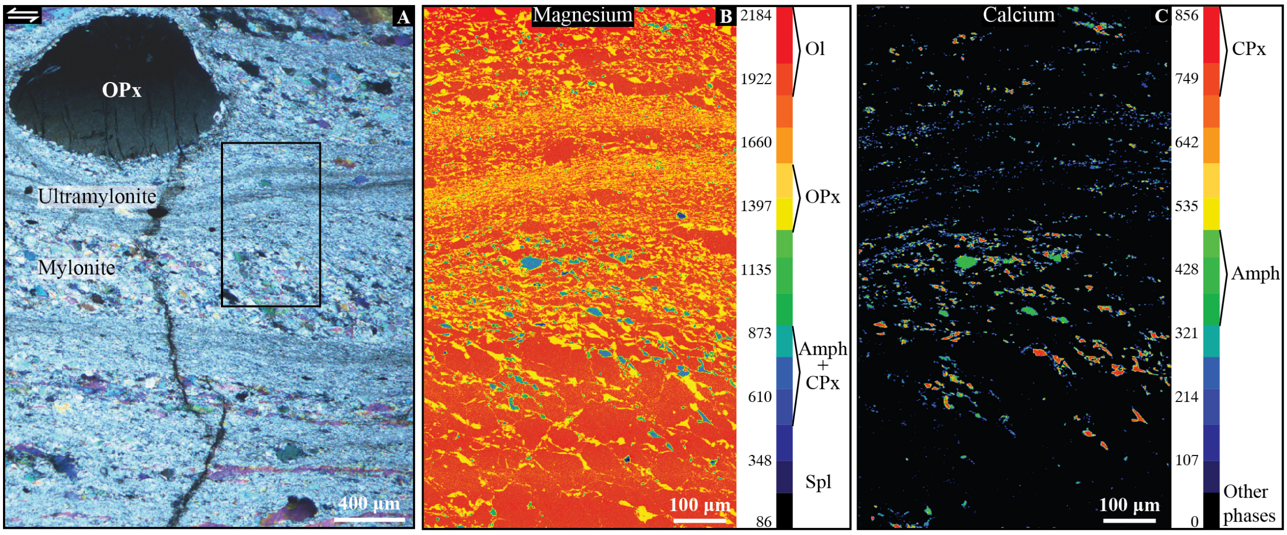

Supplementary Figure 2 | Element maps across ultramylonite layers. A) Thin section (polarized light) of a mylonitic peridotite, including ultramylonites. **B)** Distribution of magnesium (microprobe) across (ultra)mylonitic layers. Location in supplementary figure 2A. The colour coding refers to point counting. The step size is 0.8 µm and the analytical conditions are 15 kV and 10 nA on polished thin section (diamond paste of 1 µm). Ol = olivine; Amph = amphibole; CPx = clinopyroxene; Spl = Spinel. **C)** Distribution of calcium across the same area. This map highlights an enrichment in amphibole nearby ultramylonite layers. OPx = orthopyroxene.

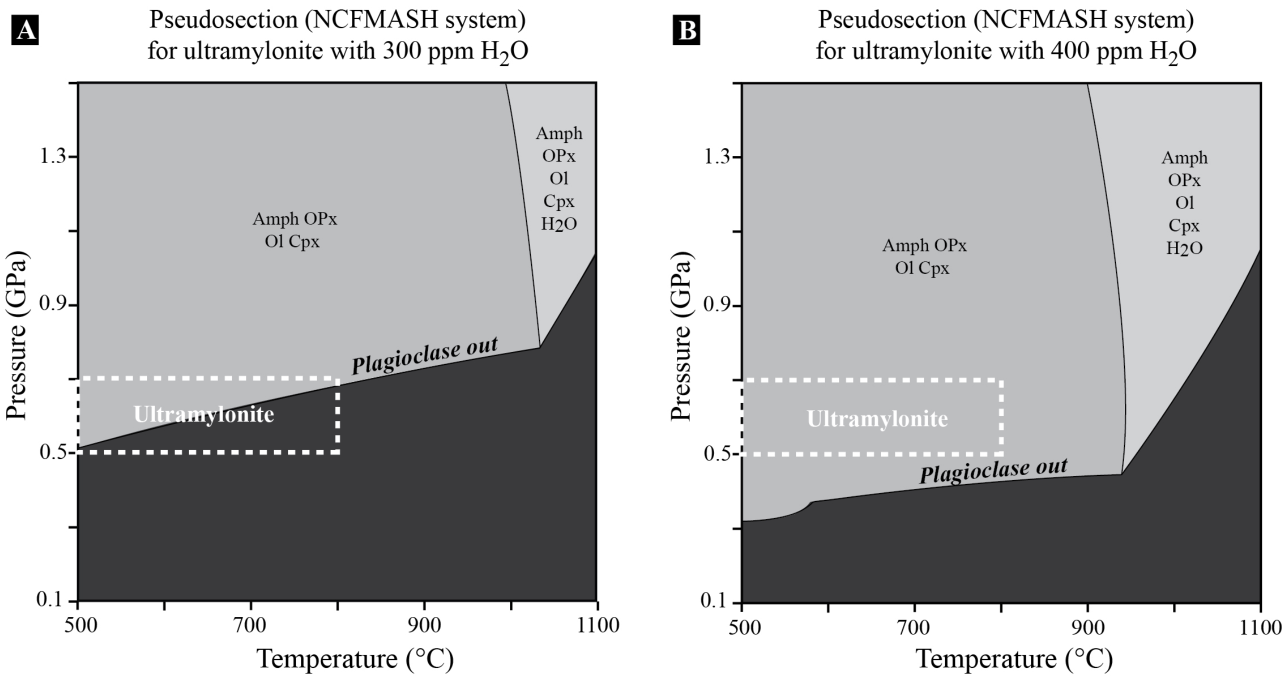

Supplementary Figure 3 | Equilibrium phase diagrams (pseudosections) for ultramylonites. A) Pseudosection with 300 ppm H_2O. **B)** Pseudosection with 400 ppm H_2O. The pseudosections were produced using Perple_X calculations in the NCFMASH (Na_2O-CaO-FeO-MgO-Al_2O_3-SiO_2-H_2O) chemical system, and based on the modal proportions that include olivine (65.9%), orthopyroxene (28.8%), amphibole (3.0%), clinopyroxene (1.5%) and spinel (0.8%). For comparison, we show the calculated conditions of deformation for the ultramylonites (dotted white rectangle). Based on the absence of plagioclase in the Ronda shear zones, our Perple_X calculations indicate a minimum of ~400 ppm H_2O in the ultramylonite layers. Amph = amphibole; OPx = orthopyroxene; Ol = Olivine; CPx = clinopyroxene.

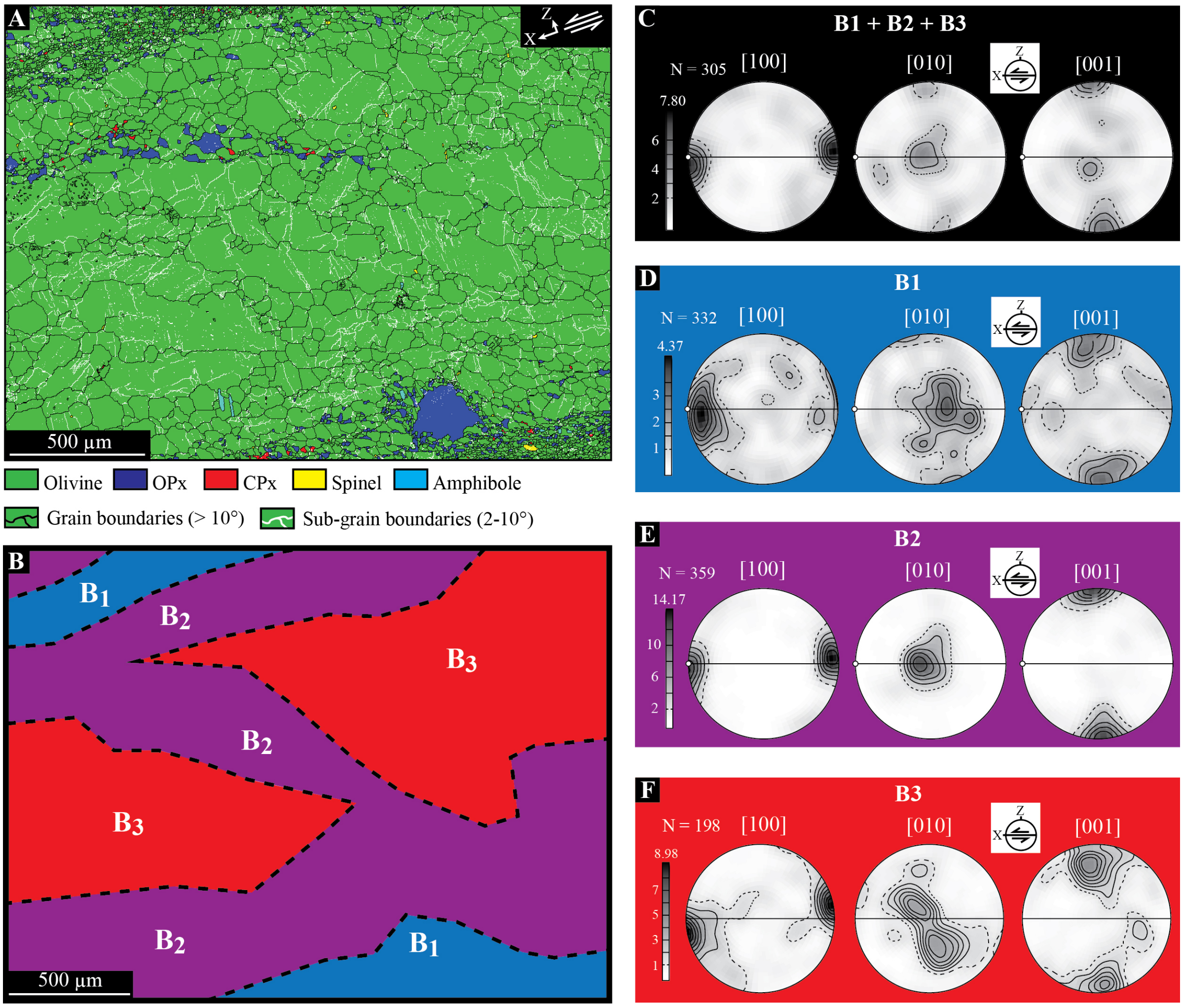

Supplementary Figure 4 | Phase distribution and olivine fabric between ultramylonites. A) EBSD map (phases) of the mylonitic layer shown in figure 5A of the paper. The step size is 1 µm and the analytical conditions are described in the methods section of the paper. While grain boundaries (black lines) are defined by correlated misorientation angles of more than 10°, the lattice tilt boundaries (white lines) are defined by correlated misorientation angles between 2° and 10°. X = shear direction; Z = pole to the shear plane. The black points within grains are non-indexed points. **B)** Sketch of the different areas highlighted in figure 5A of the paper and based on the olivine LPO distribution, including ultramylonites (B1), connected bands of strong LPO in the mylonite (B2), and isolated areas of moderate LPO between the ultramylonites (B3). **From C to F)** Olivine LPOs for each area, including the whole map (C), B1 area (D), B2 area (E) and B3 area (F). The three orthorhombic axes for olivine ([100], [010] and [001]) are shown in lower-hemisphere pole figures with respect to the shear plane (horizontal line) and shear direction (white dot). The iso-contours and grey shading (linear scale) are multiples of a uniform distribution. They were constructed using a Gaussian half-width angle of 10° and based on one point per grain. N = number of grains; OPx = orthopyroxene; CPx = clinopyroxene.

Supplementary Table 1 | Major-element compositions (in weight percent) and grain size of olivine (Ol) grains in the mylonites and ultramylonites. Por = porphyroclasts.

Oxides (%)	Ol_1	Ol_2	Ol_3	Ol_4	Ol_5	Ol_6	Ol_7	Ol_8	Ol_9	Ol_{10}	Ol_{11}	Ol_{12}
SiO_2	40.86	40.96	41.11	41.00	41.03	41.13	41.2	40.85	40.38	40.80	40.91	40.89
TiO_2	0.00	0.00	0.00	0.00	0.00	0.00	0.00	0.00	0.00	0.00	0.00	0.00
Al_2O_3	0.00	0.00	0.00	0.00	0.00	0.00	0.00	0.00	0.00	0.00	0.00	0.00
Cr_2O_3	0.00	0.03	0.01	0.01	0.01	0.00	0.00	0.00	0.00	0.00	0.00	0.01
FeO	8.03	7.82	7.69	7.80	8.39	8.30	8.25	8.34	8.19	7.95	7.96	7.79
MnO	0.12	0.12	0.11	0.12	0.13	0.13	0.12	0.13	0.12	0.12	0.12	0.12
MgO	50.32	50.32	50.31	50.16	50.32	50.72	50.65	50.51	50.66	50.87	50.59	51.36
CaO	0.02	0.03	0.06	0.02	0.00	0.00	0.01	0.02	0.00	0.00	0.02	0.00
Na_2O	0.00	0.00	0.00	0.00	0.00	0.00	0.00	0.01	0.00	0.00	0.00	0.00
NiO	0.40	0.40	0.41	0.38	0.38	0.39	0.40	0.40	0.39	0.40	0.40	0.39
Total	99.75	99.66	99.69	99.50	100.3	100.7	100.7	100.3	99.74	100.1	100.0	100.6
Grain size (μm)	250	40	25	25	80	60	300	400	Por	Por	Por	10

Oxides (%)	Ol_{13}	Ol_{14}	Ol_{15}	Ol_{16}	Ol_{17}	Ol_{18}	Ol_{19}	Ol_{20}	Ol_{21}	Ol_{22}
SiO_2	40.88	41.47	41.28	41.12	41.21	41.15	40.92	41.21	41.10	40.92
TiO_2	0.00	0.00	0.00	0.00	0.00	0.00	0.00	0.00	0.00	0.00
Al_2O_3	0.00	0.00	0.00	0.00	0.00	0.00	0.00	0.00	0.00	0.00
Cr_2O_3	0.00	0.03	0.02	0.00	0.01	0.01	0.00	0.21	0.00	0.00
FeO	7.78	7.11	7.44	8.13	8.08	7.91	8.06	7.75	7.71	8.06
MnO	0.11	0.11	0.11	0.12	0.12	0.12	0.12	0.13	0.11	0.12
MgO	51.48	50.85	50.48	50.94	51.15	50.48	50.39	50.49	50.43	50.39
CaO	0.00	0.03	0.05	0.01	0.01	0.14	0.01	0.01	0.01	0.01
Na_2O	0.00	0.00	0.00	0.00	0.00	0.00	0.00	0.01	0.00	0.00
NiO	0.41	0.43	0.39	0.40	0.42	0.39	0.40	0.39	0.42	0.4
Total	100.7	100.0	99.77	100.7	101.0	100.2	99.90	100.2	99.77	100.0
Grain size (μm)	10	100	80	150	80	30	200	30	100	200

Supplementary Table 2 | Major-element compositions (oxides in weight percent) and grain size of enstatite (En) grains in the mylonites and ultramylonites. Por = porphyroclasts.

Oxides	En_1	En_2	En_3	En_4	En_5	En_6	En_7	En_8	En_9	En_{10}	En_{11}	En_{12}
SiO_2	55.43	55.84	55.68	55.54	56.00	55.80	56.59	56.70	56.63	55.47	55.91	57.57
TiO_2	0.01	0.01	0.02	0.00	0.00	0.01	0.02	0.00	0.01	0.00	0.01	0.00
Al_2O_3	2.20	1.70	1.79	2.03	1.39	1.35	1.01	0.54	0.50	0.54	1.97	0.13
Cr_2O_3	0.71	0.48	0.56	0.61	0.27	0.27	0.16	0.04	0.07	0.09	0.75	0.05
FeO	5.49	5.61	5.52	5.71	5.62	5.62	5.62	5.69	5.30	5.85	5.41	5.55
MnO	0.13	0.14	0.15	0.15	0.14	0.14	0.14	0.12	0.12	0.12	0.14	0.14
MgO	34.34	34.81	34.90	34.91	35.25	35.25	35.51	35.73	36.07	37.10	34.89	36.00
CaO	1.05	0.71	0.54	0.55	0.51	0.53	0.33	0.27	0.24	0.26	0.51	0.09
Na_2O	0.03	0.02	0.02	0.02	0.03	0.01	0.00	0.00	0.02	0.00	0.00	0.00
K_2O	0.00	0.01	0.00	0.00	0.00	0.00	0.01	0.00	0.00	0.00	0.00	0.00
Total	99.39	99.33	99.16	99.52	99.20	98.99	99.38	99.09	98.95	99.45	99.59	99.53
Grain size (µm)	Por (rim)	Por (core)	Por (rim)	Por (core)	30	20	20	40	5	5	Por (core)	Por (rim)

Oxides	En_{13}	En_{14}	En_{15}	En_{16}	En_{17}	En_{18}	En_{19}	En_{20}	En_{21}	En_{22}	En_{23}	En_{24}
SiO_2	56.82	57.39	57.77	56.44	56.66	57.06	56.64	56.34	56.66	57.14	56.78	56.92
TiO_2	0.01	0.00	0.02	0.00	0.02	0.01	0.01	0.02	0.02	0.01	0.01	0.00
Al_2O_3	0.17	0.24	0.14	0.85	1.39	0.55	1.01	0.83	1.05	0.55	0.95	1.84
Cr_2O_3	0.03	0.04	0.05	0.16	0.30	0.08	0.15	0.12	0.17	0.09	0.14	0.80
FeO	5.73	5.80	5.76	5.96	5.66	5.45	5.57	5.86	5.64	5.57	5.61	5.67
MnO	0.14	0.13	0.15	0.15	0.13	0.12	0.13	0.14	0.15	0.16	0.14	0.16
MgO	36.94	36.00	36.19	36.41	35.42	35.98	35.34	36.03	35.56	35.79	35.68	33.71
CaO	0.06	0.08	0.09	0.27	0.40	0.28	0.39	0.33	0.47	0.30	0.31	0.49
Na_2O	0.00	0.00	0.00	0.00	0.00	0.00	0.01	0.04	0.00	0.00	0.01	0.02
K_2O	0.00	0.00	0.00	0.00	0.00	0.00	0.00	0.01	0.00	0.00	0.01	0.00
Total	99.89	99.66	100.16	100.23	99.99	99.51	99.26	99.70	99.72	99.61	99.65	99.59
Grain size (µm)	10	15	10	8	40	10	20	10	50	6	10	Por (core)

Oxides	En_{25}	En_{26}	En_{27}	En_{28}	En_{29}	En_{30}	En_{31}	En_{32}	En_{33}	En_{34}	En_{35}	En_{36}
SiO_2	57.13	56.72	57.33	57.62	56.94	57.11	57.63	57.12	57.12	57.60	57.37	57.27
TiO_2	0.01	0.00	0.01	0.00	0.01	0.01	0.00	0.02	0.01	0.00	0.00	0.01
Al_2O_3	1.75	0.42	1.08	0.45	1.30	1.98	1.46	1.19	1.15	0.39	0.67	0.60
Cr_2O_3	0.68	0.03	0.17	0.07	0.24	0.81	0.62	0.19	0.18	0.04	0.07	0.05
FeO	5.23	5.34	5.53	5.55	5.51	5.62	5.76	5.50	5.61	5.45	5.46	5.80
MnO	0.26	0.13	0.14	0.14	0.11	0.15	0.12	0.13	0.16	0.13	0.14	0.13
MgO	33.91	36.10	35.55	36.00	35.31	33.68	34.14	35.48	35.45	36.15	35.84	35.82
CaO	0.55	0.65	0.36	0.28	0.43	1.07	0.34	0.42	0.41	0.29	0.26	0.25
Na_2O	0.02	0.01	0.03	0.00	0.01	0.04	0.00	0.02	0.02	0.02	0.00	0.03
K_2O	0.02	0.00	0.00	0.00	0.00	0.00	0.00	0.00	0.00	0.00	0.00	0.01
Total	99.55	99.39	100.2	100.1	99.85	100.5	100.1	100.1	100.1	100.1	99.80	99.98
Grain size (µm)	Por (core)	8	20	35	30	Por (core)	Por (rim)	30	15	20	15	10

Supplementary Table 2 (continued)

Oxides	En_{37}	En_{38}	En_{39}	En_{40}	En_{41}	En_{42}	En_{43}	En_{44}	En_{45}	En_{46}	En_{47}	En_{48}	En_{49}
SiO_2	57.08	57.49	58.23	55.89	57.06	56.70	56.88	55.81	57.61	56.70	57.55	57.29	57.71
TiO_2	0.01	0.05	0.00	0.01	0.02	0.01	0.01	0.01	0.02	0.02	0.01	0.00	0.05
Al_2O_3	1.13	1.44	1.35	0.89	0.91	1.33	1.16	0.62	0.64	1.02	0.48	1.82	1.17
Cr_2O_3	0.22	0.36	0.31	0.18	0.21	0.29	0.22	0.08	0.09	0.16	0.05	0.69	0.31
FeO	5.51	5.46	5.98	5.72	5.33	5.52	5.48	5.73	5.42	5.51	5.36	5.85	5.50
MnO	0.14	0.18	0.10	0.14	0.11	0.14	0.15	0.12	0.14	0.14	0.13	0.09	0.26
MgO	35.49	33.97	34.56	36.57	35.46	35.20	35.43	35.92	35.41	35.13	35.90	33.71	34.33
CaO	0.36	0.55	0.61	0.30	0.29	0.52	0.39	0.26	0.27	0.38	0.21	0.60	0.45
Na_2O	0.00	0.03	0.00	0.00	0.00	0.00	0.01	0.02	0.00	0.00	0.00	0.02	0.02
NiO	0.00	0.00	0.00	0.00	0.00	0.00	0.00	0.00	0.00	0.01	0.00	0.01	0.00
Total	99.95	99.52	101.1	99.72	99.39	99.71	99.73	98.58	99.59	99.07	99.71	100.1	99.90
Grain size	10	Por (rim)	Por (rim)	20	25	40	40	8	40	30	20	Por (core)	Por (rim)

Supplementary Table 3 | Major-element compositions (oxides in weight percent) and grain size of diopside (Di) grains in the mylonites and ultramylonites. Por = porphyroclasts.

Oxides (%)	Di_1	Di_2	Di_3	Di_4	Di_5	Di_6	Di_7	Di_8	Di_9	Di_{10}	Di_{11}	Di_{12}
SiO_2	54.24	53.95	53.95	54.26	53.70	54.24	53.87	53.57	53.30	53.95	54.12	53.44
TiO_2	0.03	0.03	0.02	0.02	0.05	0.01	0.01	0.04	0.06	0.07	0.04	0.05
Al_2O_3	1.57	1.94	1.90	1.20	2.23	1.55	1.60	1.97	2.21	1.78	1.28	1.55
FeO	2.11	2.26	2.22	2.13	2.27	2.15	2.19	2.15	2.31	2.22	2.03	2.19
MnO	0.06	0.08	0.06	0.08	0.09	0.07	0.06	0.08	0.07	0.07	0.05	0.07
MgO	17.32	17.34	17.38	17.66	16.86	17.45	17.16	17.35	17.39	17.07	17.45	17.16
CaO	23.14	22.85	23.07	23.38	23.21	23.32	23.33	22.72	22.53	23.18	23.44	23.47
Na_2O	0.59	0.59	0.63	0.53	0.67	0.60	0.58	0.71	0.65	0.58	0.52	0.47
K2O	0.00	0.00	0.00	0.00	0.00	0.00	0.00	0.00	0.01	0.00	0.00	0.00
Cr_2O_3	0.55	0.72	0.65	0.39	0.81	0.55	0.55	0.89	0.99	0.70	0.33	0.44
Total	99.59	99.75	99.88	99.66	99.88	99.95	99.35	99.48	99.52	99.61	99.27	98.85
Grain size (µm)	15	30	50	8	15	5	10	200	100	25	15	25

Oxides (%)	Di_{13}	Di_{14}	Di_{15}	Di_{16}	Di_{17}	Di_{18}
SiO_2	54.14	53.27	53.12	53.96	54.10	53.55
TiO_2	0.04	0.02	0.01	0.03	0.01	0.02
Al_2O_3	1.36	1.73	1.93	1.69	1.29	1.82
FeO	2.20	2.26	2.30	2.29	2.13	2.19
MnO	0.06	0.07	0.07	0.07	0.07	0.05
MgO	17.23	17.38	17.33	17.36	17.55	17.22
CaO	23.34	23.33	23.00	23.29	23.81	23.23
Na_2O	0.49	0.51	0.54	0.59	0.51	0.65
K2O	0.00	0.01	0.00	0.00	0.00	0.00
Cr_2O_3	0.38	0.65	0.73	0.59	0.42	0.68
Total	99.25	99.22	99.02	99.86	99.89	99.42
Grain size (µm)	15	10	60	10	10	80

Supplementary table 4 | Major-element compositions (oxides in weight percent) and grain size of spinel (Spl) grains in the mylonites and ultramylonites. Por = porphyroclasts.

Oxides (%)	Spl_1	Spl_2	Spl_3	Spl_4	Spl_5	Spl_6	Spl_7	Spl_8	Spl_9	Spl_{10}	Spl_{11}	Spl_{12}
SiO_2	0.05	0.20	0.04	0.01	0.01	0.00	0.03	0.04	0.05	0.01	0.84	0.02
TiO_2	0.04	0.02	0.01	0.01	0.01	0.07	0.03	0.03	0.08	0.08	0.04	0.02
Al_2O_3	22.13	20.62	30.47	26.08	27.20	20.48	28.38	27.71	24.24	23.69	24.68	24.12
Cr_2O_3	43.16	41.45	36.02	41.28	39.80	45.57	36.96	36.07	41.91	42.48	39.30	42.71
V2O3	0.15	0.16	0.18	0.18	0.19	0.15	0.17	0.16	0.15	0.16	0.15	0.19
FeO	17.19	19.84	17.60	14.78	16.85	16.05	17.92	18.06	15.72	15.69	17.12	15.73
Fe2O3	6.02	7.38	2.77	4.43	3.71	5.61	4.46	5.56	5.16	5.51	4.21	4.63
MnO	0.22	0.28	0.21	0.19	0.21	0.21	0.22	0.22	0.20	0.20	0.21	0.20
MgO	11.91	9.88	12.13	13.90	12.58	12.45	11.83	11.54	13.13	13.10	12.59	13.03
NiO	0.11	0.10	0.08	0.12	0.10	0.11	0.09	0.11	0.12	0.12	0.10	0.11
ZnO	0.25	0.24	0.49	0.17	0.25	0.13	0.42	0.39	0.13	0.16	0.17	0.19
Total	101.2	100.2	100.0	101.2	101.0	100.8	100.5	99.90	100.9	101.2	99.41	100.9
Grain size (µm)	400	15	20	Por	80	Por	10	10	150	300	80	150

Oxides (%)	Spl_{13}	Spl_{14}	Spl_{15}	Spl_{16}	Spl_{17}	Spl_{18}
SiO_2	0.01	0.07	0.03	0.00	0.00	0.02
TiO_2	0.02	0.02	0.05	0.06	0.04	0.05
Al_2O_3	26.10	30.72	21.46	21.28	23.35	21.61
Cr_2O_3	39.82	35.14	45.03	44.93	42.89	44.91
V2O3	0.19	0.19	0.16	0.17	0.17	0.17
FeO	16.32	16.24	15.33	16.42	16.00	15.49
Fe2O3	4.91	3.87	5.32	5.28	5.12	5.22
MnO	0.21	0.19	0.20	0.22	0.20	0,.20
MgO	12.75	13.18	13.05	12.30	12.75	12.96
NiO	0.12	0.12	0.12	0.10	0.11	0.11
ZnO	0.22	0.27	0.13	0.15	0.17	0.14
Total	100.7	100.0	100.9	100.1	100.8	100.9
Grain size (µm)	100	10	Por	10	200	Por

Supplementary Table 5 | Major-element compositions (oxides in weight percent) of amphibole grains (Amp) in the mylonites and ultramylonites. Por = porphyroclasts.

Oxides (%)	Amp1	Amp2	Amp3	Amp4	Amp5	Amp6	Amp7	Amp8	Amp9	Amp10
**SiO_2**	44.81	43.92	44.28	43.81	44.00	44.16	44.27	43.89	44.40	43.62
**TiO_2**	0.34	0.44	0.40	0.21	0.21	0.21	0.36	0.49	0.42	0.50
**Al_2O_3**	13.11	13.42	13.23	13.05	13.01	12.85	12.99	13.39	12.64	13.22
**Cr_2O_3**	1.56	2.17	1.90	2.11	2.04	2.24	1.83	1.91	1.58	1.91
**Fe_2O_3**	3.73	3.95	3.91	3.82	3.75	3.86	4.08	3.91	4.22	4.22
**FeO**	0.00	0.00	0.00	0.00	0.00	0.00	0.00	0.00	0.00	0.00
**MnO**	0.06	0.05	0.04	0.05	0.06	0.06	0.01	0.04	0.04	0.05
**MgO**	19.02	18.42	18.73	18.93	18.90	18.76	18.75	18.19	18.68	18.37
**CaO**	12.04	11.99	12.03	11.95	11.93	11.87	12.14	12.04	11.96	12.10
**Na_2O**	2.77	2.88	2.85	3.01	3.24	3.28	3.12	2.85	2.88	2.94
**K_2O**	0.09	0.09	0.08	0.08	0.07	0.07	0.17	0.21	0.19	0.20
**H_2O**	2.13	2.12	2.12	2.11	2.11	2.12	2.12	2.11	2.11	2.11
**Total**	99.66	99.44	99.58	99.12	99.34	99.47	99.84	99.03	99.12	99.25

Supplementary Table 6 | Pressure (P in kbar) and temperature (T in °C) data estimated in the mylonites and ultramylonites based on the major-elements compositions and geothermobarometers. Each number refers to the phase number shown in previous tables (from tables S1 to S5). The temperature data are summarized in the figure 3D of the paper. (1) = Ca in OPx at 5 kbar[1]; (2) = Ca in OPx at 8 kbar[1]; (3) = Al and Cr in OPx[2]; (4) = Two-pyroxene thermometer at 5 kbar[1] (TBKN); (5) = two-pyroxene thermometer at 8 kbar[1]. (6) = Olivine-spinel thermometer[3]. (7) = Two-pyroxene barometer[4].

Phase(s)	En_1	En_2	En_3	En_4	En_5	En_6	En_7	En_8	En_9	En_{10}	En_{11}	En_{12}	En_{13}	En_{14}	En_{15}	En_{16}	En_{17}	En_{18}
T (1)	1016.9	923.4	866.7	871.6	855.2	865.5	777.2	742.0	726.0	740.5	856.3	596.7	559.5	586.0	595.2	742.5	811.3	752.1
T (2)	1032.5	937.8	880.5	885.4	868.9	879.3	789.9	754.2	738.1	752.8	870.0	607.3	569.6	596.4	605.7	754.8	824.4	764.4
T (3)	932.05	823.6	858.1	873.9	723.8	713.7	683.9	613.3	610.9	510.9	950.2	624.4	537.6	614.1	615.1	617.4	754.1	635.6

Phase(s)	En_{19}	En_{20}	En_{21}	En_{22}	En_{23}	En_{24}	En_{25}	En_{26}	En_{27}	En_{28}	En_{29}	En_{30}	En_{31}	En_{32}	En_{33}	En_{34}	En_{35}	En_{36}
T (1)	804.5	776.2	838.4	759.6	766.5	847.3	869.6	905.8	790.6	750.4	821.5	1018.0	780.4	818.9	812.2	754.8	734.8	731.0
T (2)	817.5	788.7	851.9	772.1	779.0	860.8	883.4	920.0	803.5	762.8	834.7	1033.6	793.2	832.1	825.3	767.2	747.0	743.1
T (3)	691.0	616.2	683.3	644.6	672.3	1054.9	1009.9	714.2	641.7	746.1	976.4	1047.4	978.6	720.6	715.4	619.9	654.0	627.2

Phase(s)	En_{37}	En_{38}	En_{39}	En_{40}	En_{41}	En_{42}	En_{43}	En_{44}	En_{45}	En_{46}	En_{47}	En_{48}	En_{49}	Di_1/En_{26}	Di_2/En_{27}	Di_3/En_{32}	Di_4/En_{34}	Di_5/En_{35}
T (1)	791.5	869.5	887.6	759.7	753.9	858.0	804.7	737.8	742.9	800.3	708.4	886.5	830.5					
T (2)	804.4	883.3	901.6	772.2	766.3	871.7	817.7	750.0	755.2	813.3	720.3	900.5	843.8					
T (3)	730.6	883.0	840.4	601.3	728.8	760.8	724.7	586.5	690.7	709.4	647.7	1017.2	841.9					
T (4)														804.6	852.3	806.7	769.2	759.9
T (5)														809.1	857.1	811.3	773.5	764.3

Phase(s)	Di_6/En_{36}	Di_7/En_{37}	Di_8/En_{42}	Di_9/En_{41}	Di_{10}/En_{40}	Di_{11}/En_{47}	Di_{12}/En_{45}	Di_{13}/En_{44}	Di_{14}/En_{10}	Di_{15}/En_6	Di_{16}/En_{18}	Di_{17}/En_{20}	Di_{18}/En_{21}	Ol_1/SPl_1	Ol_2/SPl_2	Ol_3/SPl_2	Ol_4/SPl_2	Ol_5/SPl_3
T (4)	766.8	740.2	827.8	870.4	785.6	742.8	717.5	768.9	752.2	800.8	786.7	866.4	743.1					
T (5)	771.1	744.5	832.4	875.2	790.1	747	721.6	773.3	756.5	805.4	791.2	871.3	747.3					
T (6)														749.0	656.0	652.0	657.0	696.0

Phase(s)	Ol_4/SPl_2	Ol_5/SPl_3	Ol_6/SPl_3	Ol_7/SPl_4	Ol_8/SPl_5	Ol_9/SPl_6	Ol_{10}/SPl_6	Ol_{11}/SPl_6	Ol_{12}/SPl_7	Ol_{13}/SPl_8	Ol_{14}/SPl_9	Ol_{15}/SPl_{10}	Ol_{15}/SPl_{11}	Ol_{16}/SPl_{12}	Ol_{17}/SPl_{13}	Ol_{18}/SPl_{14}	Ol_{19}/SPl_{15}	Ol_{20}/SPl_{16}	Ol_{21}/SPl_{17}	Ol_{22}/SPl_{18}
T (6)	657.0	696.0	691.0	821.0	750.0	811.0	800.0	802.0	660.0	641.0	745.0	764.0	726.0	796.0	743.0	707.0	827.0	780.0	772.0	822.0

Phase(s)	Di_1/En_{26}	Di_2/En_{27}	Di_3/En_{32}	Di_4/En_{34}	Di_5/En_{35}	Di_6/En_{36}	Di_7/En_{37}	Di_8/En_{42}	Di_9/En_{41}	Di_{10}/En_{40}	Di_{11}/En_{47}	Di_{12}/En_{45}	Di_{13}/En_{44}	Di_{14}/En_{10}	Di_{15}/En_6	Di_{16}/En_{18}	Di_{17}/En_{20}	Di_{18}/En_{21}
P (7)	4.8	9.0	6.9	8.8	10.6	10.7	6.5	5.9	11.8	9.0	10.8	7.7	9.6	9.2	4.9	8.9	6.1	5.1

Supplementary References

1. Brey, G. P., & Köhler, T. Geothermobarometry in four-phase lherzolites II. New thermobarometers, and practical assessment of existing thermobarometers. *J. Petrol.* **31,** 1353-1378 (1990).
2. Witt-Eickschen, G. & Seck, H. A. Solubility of Ca and Al in orthopyroxene from spinel peridotite: an improved version of an empirical geothermometer. *Contrib. Min. Petrol*. **106,** 431-439 (1991).
3. Jianping, L., Kornprobst, J., Vielzeuf, D. & Fabriès, J. An improved experimental calibration of the olivine-spinel geothermometer. *Chinese J. Geochem.* **14,** 68-77 (1995).
4. Putirka, K. D. Thermometers and barometers for volcanic systems. *Rev. Min. Geochem*. **69,** 61-120 (2008).
